# Risk factors for adverse reactions from contrast agents for computed tomography

**DOI:** 10.1186/1472-6947-13-18

**Published:** 2013-01-30

**Authors:** Daiki Kobayashi, Osamu Takahashi, Takuya Ueda, Gautam A Deshpande, Hiroko Arioka, Tsuguya Fukui

**Affiliations:** 1 Division of General Internal Medicine, Department of Medicine, St. Luke’s International Hospital, Tokyo, Japan; 2 Division of General Internal Medicine, Department of Medicine, St. Luke’s International Hospital, 9-1 Akashi-cho, Chuo-ku, Tokyo, 104-8560, Japan; 3 Department of Radiology, St. Luke’s International Hospital, 9-1 Akashi-cho, Chuo-ku, Tokyo, 104-8560, Japan; 4 St. Luke’s Life Science Institute, St. Luke’s International Hospital, 9-1 Akashi-cho, Chuo-ku, Tokyo, 104-8560, Japan; 5 Department of Internal Medicine, University of Hawaii, Honolulu, HI, Hawaii

## Abstract

**Background:**

Symptoms of an adverse reaction to contrast agents for computed tomography are diverse ranging, and sometimes serious. The goal of this study is to create a scoring rule to predict adverse reactions to contrast agents used in computed tomography.

**Methods:**

This was a retrospective cohort study of all adult patients undergoing contrast enhanced CT scan for 7 years. The subjects were randomly divided into either a derivation or validation group. Baseline data and clinically relevant factors were collected from the electronic chart. Primary outcome was any acute adverse reactions to contrast media, observed for during 24 hours after administration. All potential candidate predictors were included in a forward stepwise logistic regression model. Prediction scores were assigned based on β coefficient. A receiver operating characteristic (ROC) curve was drawn, and the area under the curve (AUC) and incidence of acute adverse reactions at each point were obtained. The same process was performed in the validation group.

**Results:**

36,472 patients underwent enhanced CT imaging: 20,000 patients in the derivation group and 16,472 in the validation group. A total of 409 (2.0%, 95% CI:1.9-2.3) and 347 (2.1%, 95% CI:1.9-2.3) acute adverse reactions were seen in the derivation and validation groups. Logistic regression analysis revealed that prior adverse reaction to contrast agents, urticaria, an allergic history to drugs other than contrast agents, contrast agent concentration >70%, age <50 years, and total contrast agent dose >65 g were significant predictors of an acute adverse reaction. AUC was 0.70 (95% CI:0.67-0.73) and 0.67 (95% CI:0.64-0.70) in the derivation and validation groups.

**Conclusions:**

We suggest a prediction model consisting of six predictors for acute adverse reactions to contrast agents used in CT.

## Background

Computed tomography (CT) imaging has rapidly become a commonplace diagnostic tool due to its utility in a wide range of diseases. A national survey in the United States estimates that approximately 70 million CT scan are performed per year [1]. In Japan, the number of CT scans performed is not only substantially higher than in other countries, but continues to increase [2,3]. Previous study reported the number of CT scans performed was about 36,550,000 times a year [4]. As the number of CT scanners increases, the number of diagnostic CT imaging studies increases as well as their side effects, including iatrogenic cancer [5] and adverse events from iodine-containing contrast agents [6,7].

Previous studies have shown that the incidence of acute adverse reactions to contrast agents is approximately 2-3% with low-osmolarity contrast agents (LOCAs) [8,9]. Symptoms of an adverse reaction to contrast agents are diverse ranging from flushing, pruritus, uriticaria, and angioedema [10], with more severe side effects including hypotension, loss of consciousness, to potentially life-threatening bronchospasm and airway obstruction [11]. These adverse events are hypersensitivity reaction, and classified into allergic reactions and non-allergic reactions [12], the former, dependent on [13], and the latter, independent on dose and infusion rate [14]. In this study, we focused on all kinds of adverse events for contrast agent for CT scans.

Risk factors for these adverse effects have been reported in previous literature, with a history of immediate adverse reaction to contrast agents being the most significant [15]. A history of allergy-mediated disease, including asthma, atopic dermatitis, and urticaria, is also considered a significant risk factor [16]. However, no prior study has examined to quantitatively adverse effects of contrast agents. A prediction rule may facilitate the pre- and post-imaging management of patients requiring contrast enhanced CT imaging. For instance, physicians can consider whether they prescribe pre-medications for high risk patients based on the rule. In other examples, physicians may choose performing CT scans without contrast agent. Finally, physicians can evaluate which patients they should observe carefully and for a long time after CT scans. The goal of this study is to evaluate risk factors for the incidence of adverse reactions to contrast agents based on relevant patient demographic and clinical factors.

## Methods

A retrospective cohort study of all adult patients who underwent contrast enhanced CT imaging with intravenous contrast agents from April 2004 through March 2011 was conducted at St. Luke's International Hospital, a large community hospital in Tokyo, Japan. All potential prognostic prediction parameters were collected prior to imaging and were based on previous studies, as well as physician-driven clinical relevance. Parameters were composed of patients’ 1) demographic data, 2) administered contrast agents, 3) allergic history, 4) medical history, and 5) laboratory test results. If patients underwent more than one CT scan during the study period, only the most recent data were included.

Demographic data included gender and age. Data on contrast agents included type of the agent, contrast agent concentration, and total contrast agent dose [17]. Allergic history included any history of antibiotics, or any other drug [10,18]. Medical history included atopic dermatitis, urticaria, asthma, hypertension, diabetes, and dyslipidemia [10,16] (Table 1). Laboratory test results were collected by routine blood draw.

**Table 1:** Check list of baseline parameters

Gender	Allergic History
Age	For radio contrast, antibiotics, and any drugs.
Contrast agent concentration	Medical history
Total contrast agent dose	Atopic dermatitis, asthma, urticaria, diabetes, hypertension, dyslipidemia
	Laboratory values
	BUN*, Cre†, Na, K, Cl, T-bil‡, AST§, ALT** , LDH††, Glu‡‡, HgbA1c§§, WBC***, Hgb†††, Plt‡‡‡,

BUN*: Blood urea nitrogen. Cre†: Creatinine. T-bil‡ Total-bilirubin. AST§: Aspartate aminotransferase. ALT**: Alanine aminotransferase. LDH††: Lactate dehydrogenase. Glu‡‡: Glucose. HgbA1c§§: Hemoglobin A1c. WBC***: White Blood Cell. Hgb†††: Hemoglobin. Plt‡‡‡: Platelet.

Adverse reactions to contrast agents are defined based on the previous study [19]. Acute reactions which occur immediately during the injection of the contrast agents up to one hour afterwards, were observed and documented by a trained radiologist or nurse in charge of the examination for several hours after imaging. Non-acute (delayed) reactions occur more than one hour after the injection of the contrast agent. After discharge, patients were followed up at home for any reactions occurring within 24 hours of contrast administration by self reporting. Albeit infrequent, severe adverse reactions involving prolonged hypertension, angina, ventricular fibrillation, based on the previous study [20]. We analyzed all kinds of these adverse reactions.

### Statistical analysis

Descriptive statistics were employed to characterize subjects’ baseline data. In order to facilitate the use of the prediction rule in the clinical setting, continuous values were categorized into groups. Following the methodology of previous studies, laboratory test results and continuous values were dichotomized based on average values [21]. We randomly divided approximately 2/3 of patients into derivation group and others into a validation group. Randomization was performed by computer based random number table.

Univariate analysis was performed to investigate the relationship between collected data and adverse reactions. In order to select final prognostic predictors, all candidate predictors for which p-value was <0.2 in univariate analysis, as well as other clinically important variables, were included in a forward stepwise logistic regression model, with a subsequent p-value of 0.05 required for inclusion in the final model. Scores for each predictor were obtained based on the beta value from the final prediction model; total scores were calculated respectively. We decided the variable which had minimum β coefficient as a predict point 1 and the predict points of other variables based on the ratio of β coefficient of each variable to minimum variable. In order to compare the incidence of adverse reactions, patients were divided into groups according to total score. A receiver operator characteristic (ROC) curve was then drawn, and the area under the curve (AUC) and incidence of adverse reaction at each point were obtained.

For validation, the same process was performed in the derivation group. In addition, we calculated sensitivity, specificity, positive and negative likelihood ratio at several scoring cut-off points.

All analyses were conducted using SPSS software package version 15.0 (IBM, Tokyo, Japan), except 95% confidence intervals (CI) which were based on an exact binominal [22] using Stata version 10 (STATA Corp., College Station, USA). Ethical approval was obtained from the Research Ethics Committee of St. Luke’s International Hospital, Tokyo, Japan.

## Results

CT imaging with contrast agents was performed on 36,472 patients between April 1, 2004 and March 31, 2011. Of these, 20,000 patients were assigned to the derivation group and 16,472 patients to the validation group.

Table 2 shows patients characteristics in both groups. There were 409 (2.0%; 95% CI, 1.9-2.3) adverse reactions in the derivation group and 347 (2.1%; 95% CI, 1.9-2.3). Severe reactions, such as shock, hypotension, desaturation, and airway obstruction were observed in 9 cases (0.0005%; 95% CI, 0.0002-0.0009) in the derivation group and 14 (0.0008%; 95% CI, 0.0005-0.0014) in the validation group. The most frequent reaction was nausea and/or vomiting at 241 occurrences (31.8%; 95% CI, 28.6-35.3), followed by rash at 189 (25%; 95% CI, 21.9-28.2), and coughing or sneezing at 60 (7.9%; 95% CI, 6.1-10.1). Several patients had multiple symptoms simultaneously. All patients were prescribed non-ionic, low-osmolar contrast agents (iopamidol, iohexol, ioversol or iomeprol).

**Table 2:** Demographic and Clinical Characteristics

	**Derivation group (n = 20,000)**		**Validation group (n = 16,472)**	
Acute adverse reaction, n (%)	409	(2.0)	347	(2.1)
Severe reaction, n (%)	9	(0.1)	14	(0.1)
Male, n (%)	10396	(52.0)	8506	(51.6)
Age, mean (SD), year	58.3	(16.6)	58.3	(16.7)
Contrast agent concentration, mean (SD),%	65.2	(6.4)	65.2	(6.2)
Total contrast agent dose, mean (SD), g	62.5	(10.4)	62.5	(9.1)
Allergic History				
For radio contrast, n (%)	464	(2.3)	389	(2.4)
For antibiotics, n (%)	589	(2.9)	498	(3.0)
For any drugs, n (%)	1544	(7.7)	1354	(8.2)
Medical history				
Atopic dermatitis, n (%)	256	(1.3)	212	(1.3)
Asthma, n (%)	148	(0.7)	128	(0.8)
Urticaria, n (%)	1159	(5.8)	859	(5.5)
Diabetes, n (%)	3929	(19.6)	3209	(19.5)
Hypertension, n (%)	5712	(28.6)	4749	(28.7)
Dyslipidemia, n (%)	3242	(16.2)	2730	(16.6)
Laboratory values				
BUN*, mean (SD), mg/dl	15.5	(8.4)	15.5	(8.5)
Cre† , mean (SD), mg/dl	0.9	(0.9)	0.8	(0.8)
Na, mean (SD), mEq/L	140.0	(3.3)	140.0	(3.3)
K, mean (SD), mEq/L	4.1	(0.5)	4.1	(0.5)
Cl, mean (SD), mEq/L	104.9	(3.7)	104.9	(3.7)
T-bil‡, mean (SD), mg/dl	0.8	(1.0)	0.8	(1.3)
AST§, mean (SD), IU/L	40.7	(264.6)	39.4	(140.0)
ALT**, mean (SD), IU/L	35.2	(137.2)	34.5	(109.1)
LDH††, mean (SD), IU/L	233.2	(475.1)	232.4	(337.7)
Glu‡‡, mean (SD), mg/dl	120.9	(47.6)	120.5	(48.2)
HgbA1c§§, mean (SD),%	5.6	(1.2)	5.6	(1.2)
WBC***, mean (SD), ×10^3^ /μl	7.3	(5.0)	7.3	(4.7)
Hgb†††, mean (SD), g/dl	12.9	(2.1)	12.9	(2.1)
Plt‡‡‡, mead (SD), ×10^3^ /μl	235.5	(86.4)	235.5	(84.2)

BUN*: Blood urea nitrogen. Cre†: Creatinine. T-bil‡ Total-bilirubin. AST§: Aspartate aminotransferase. ALT**: Alanine aminotransferase. LDH††: Lactate dehydrogenase. Glu‡‡: Glucose. HgbA1c§§: Hemoglobin A1c. WBC***: White Blood Cell. Hgb†††: Hemoglobin. Plt‡‡‡: Platelet.

**Figure 1:**
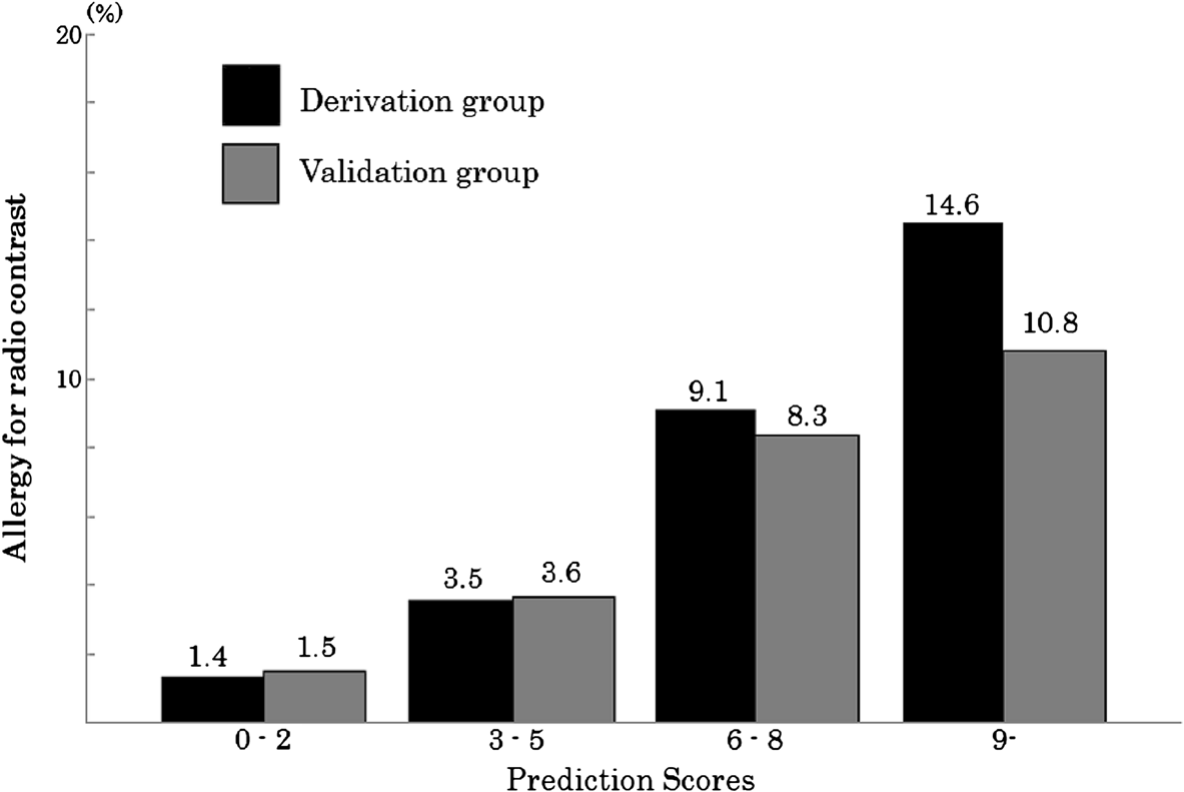
Adverse reaction for radio contrast on the Logistic Regression Model.

**Figure 2:**
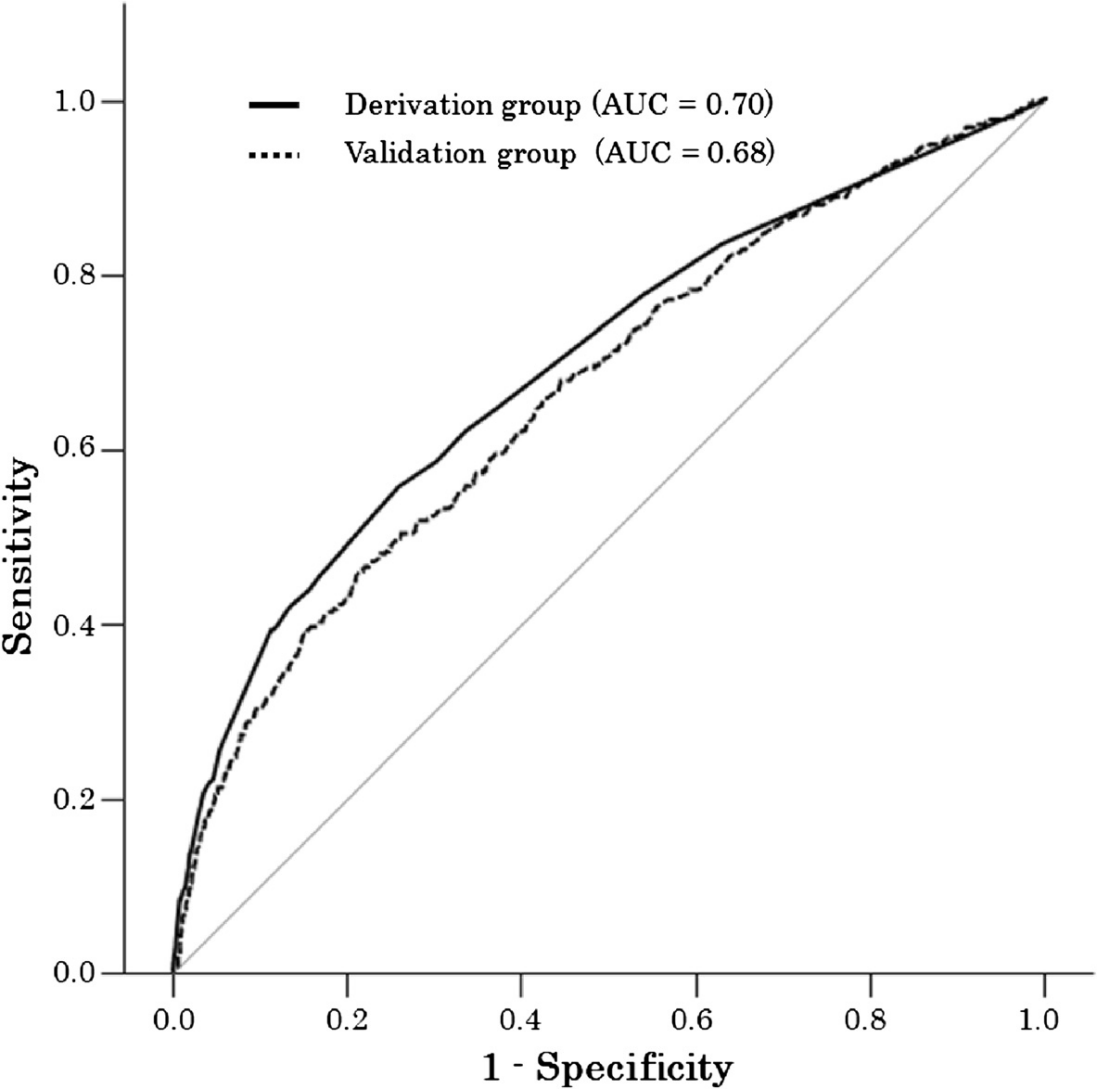
ROC curve of Radio contrast adverse reaction prediction scores.

Logistic regression analysis was constructed with all of the candidate predictors which were significant on univariate analysis. Adverse reaction history for contrast agents (odds ratio [OR], 7.1; 95% CI, 5.2-9.7), urticaria (OR 2.7; 95% CI, 2.0-3.6), allergic history to drugs other than contrast agents (OR 1.9; 95% CI, 1.5-2.6), contrast agent concentration >70% (OR 1.9; 95% CI, 1.5-2.4), age <50 years (OR 1.8; 95% CI, 1.4-2.2), and total contrast agent dose > 65 g (OR 1.4; 95% CI, 1.1-1.7) met inclusion criteria. Table 3 shows the final model of prognostic predictors. It was based on assigning predict points to each patient and determining the sum. A prediction model with a maximum score of 17 points was derived. Total scores of 0–2 point, 3–5 points, 6–8 points, and ≥9 points, were associated with adverse reaction rates of 1.4% (95% CI, 1.2-1.5), 3.5% (95% CI, 2.9-4.2), 9.1% (95% CI, 6.5-12.2), and 14.6% (95% CI, 10.1-20.0), respectively (Figure 1). We calculated the sum of scores for each patient and drew an ROC curve (Figure 2). The AUC of this prediction rule was 0.70 (95% CI, 0.67-0.73). For severe adverse reaction to contrast agent, we were not able to evaluate prediction model, because the incidence was quite low.

**Table 3:** **Predict point**’**s decision with Logistic Regression**

	**β coefficient**	**95% CI**	**Odds Ratio**	**p-value**	**Predict Point**
Adverse reaction history for radio contrast	1.96	1.64 - 2.27	7.07	< 0.001	7
Urticaria	0.98	0.70 - 1.27	2.67	< 0.001	3
Allergic history for any drugs	0.67	0.38 - 0.96	1.95	< 0.001	2
Contrast agent concentration over 70 (%)	0.63	0.38 - 0.88	1.88	< 0.001	2
Age under 50 years	0.57	0.36 - 0.77	1.76	< 0.001	2
Total contrast agent dose over 65 (g)	0.31	0.07 - 0.55	1.36	0.011	1

We conducted validation of the derived prediction model with 16,472 subjects in the validation group. Adverse reaction rates were 1.5% (95% CI, 1.3-1.7), 3.6% (95% CI, 3.0-4.4), 8.3% (95% CI, 5.7-11.8), and 10.8% (95% CI, 6.5-16.5), respectively, for each scoring group (Figure 1). ROC curve was also drown with the validation group and the AUC was 0.67 (95% CI; 0.64-0.70) (Figure 2) and was similar to data from the derivation group. Sensitivity, specificity, and positive and negative likelihood ratios at each cut-off point were calculated and are shown in Table 4.

**Table 4:** Sensitivity, specificity, positive and negative likelihood ratio in each cut off points

**Cut off point**	**Sensitivity (%)**	**Specificity (%)**	**Positive likelihood ratio**	**Negative likelihood ratio**
1	79.6	59.3	1.96	0.34
3	45.5	79.9	2.26	0.68
6	17.1	97.1	5.9	0.85
9	7.6	99.1	8.4	0.93

## Discussion

In this study, we propose a prediction model for estimating the incidence of all kinds of adverse reaction in patients undergoing contrast enhanced CT imaging. The six predictors identified by multivariate logistic regression analysis were an adverse reaction history to contrast agents, urticaria, allergic history to any other drug, contrast agent concentration in the administered agent, age, and total administered iodine dose. AUC demonstrated acceptable accuracy (AUC = 0.70, p < 0.001, 95% CI: 0.67-0.73).

This is the first study to propose a model to quantitatively estimate risk of adverse reactions to contrast agents. Predictors in our model are consistent with previously reported risk factors, such as a history of adverse reaction to contrast agents or allergy to other drugs [23,24]. Atopic individuals, such as those with asthma, dermatitis, and urticaria, have also been considered to be at higher risk for adverse events after contrast agents administration [10]. After quantitative analysis, urticaria appears to be the most significant risk factor based on odds ratio. In addition, while we know of no previous study evaluating patient’s age in association with adverse reactions, one previous study demonstrated that older patients may have stronger reactions to histamine [25]. Finally, the dose of administered iodine has been shown to be associated with iodine allergy [26] ,while chemotoxic reactions are known to be dependent on dose and infusion rate [13].

Several previously reported risk factors, including asthma and type of contrast agents, were also considered in our study, but were consequently not included among our final predictors. Previous literature suggested that well-controlled asthma patients may not continue to be at risk for contrast agents reactions although un-controlled patients are at risk [16]. In our study, in order to facilitate clinical utility of the model, we did not differentiate between well-controlled and uncontrolled asthma patients. In addition, in Japan, patients with a history of asthma are often considered poor candidates for receiving contrast, which may be avoided at the discretion of the treating radiologist at our institution. Due to treatment differences and the potential for a priori exclusion, asthma might have escaped inclusion as a predictor in our study. Similarly, osmolarity of contrast agents has been shown to be associated with adverse reactions [27]. In our study, all patients received low osmolar contrast agents, of which there was little difference in contrast agent concentration between the different types, precluding evaluation of higher osmolar agents.

There were some limitations in our study. First, our prediction model was not precise to predict adverse reaction [28]. However, at a scoring cut-off of 6 and 9, the specificity was 97.1% and 99.1%, respectively. These high specificities are useful in predicting which individuals may be at high-risk of having an adverse reaction and scoring can be done pre-imaging, thus facilitating appropriate observation periods and/or pre-procedure prophylactic therapy with steroids or antihistamines [27,29]. Second, our study was conducted only in one hospital, at which the majority of patients were Japanese. Although both large in scale and validated, prospective studies at multiple centers and with heterogeneous populations are needed in the future to further refine the model.

## Conclusion

We propose a validated prediction model for adverse reactions to contrast agents consisting of six predictors— allergic history to contrast agents, urticaria, history of previous allergy to drugs other than contrast agents, contrast agent concentration > 70%, age <50 years old, and a total contrast agent dose >65 g. Using the scoring model, this set of predictors is easy to calculate in the clinical setting and may facilitate appropriate referrals for enhanced imaging by outpatient physicians, as well as management of high-risk patients by radiologists and inpatient physicians.

## Abbreviations

ROC: Receiver operating characteristic; AUC: Area under the curve; LOCAs: Low-osmolarity contrast agents; CI: Confidence intervals; OR: Odds ratio; BUN: Blood urea nitrogen; Cre: Creatinine; T-bil: Total-bilirubin; AST: Aspartate aminotransferase; ALT: Alanine aminotransferase; LDH: Lactate dehydrogenase; Glu: Glucose; HgbA1c: Hemoglobin A1c; WBC: White Blood Cell; Hgb: Hemoglobin; Plt: Platelet.

## Competing interests

All authors have no competing interests.

## Authors’ contributions

DK conducted this study, decided study design, analyzed data, and drafted the manuscript. OT contributed to study design decision, performing data analysis and making manuscript. TU, GD, and HA checked study design, reviewed manuscript and contribute to discussion. TF organized this study and contributed to discussion. All authors have read and approved the final manuscript.

## Pre-publication history

The pre-publication history for this paper can be accessed here:

http://www.biomedcentral.com/1472-6947/13/18/prepub
